# The hidden RNA code: implications of the RNA epitranscriptome in the context of viral infections

**DOI:** 10.3389/fgene.2023.1245683

**Published:** 2023-08-01

**Authors:** Diana Roberta Ribeiro, Alexandre Nunes, Daniela Ribeiro, Ana Raquel Soares

**Affiliations:** Department of Medical Sciences, iBiMED–Institute of Biomedicine, University of Aveiro, Aveiro, Portugal

**Keywords:** RNA epitranscriptome, virus-host cell interactions, host antiviral response, coding-RNAs, non-coding RNAs

## Abstract

Emerging evidence highlights the multifaceted roles of the RNA epitranscriptome during viral infections. By modulating the modification landscape of viral and host RNAs, viruses enhance their propagation and elude host surveillance mechanisms. Here, we discuss how specific RNA modifications, in either host or viral RNA molecules, impact the virus-life cycle and host antiviral responses, highlighting the potential of targeting the RNA epitranscriptome for novel antiviral therapies.

## Introduction

Viruses are completely dependent on their host cells’ machinery to replicate and form new infectious virus particles, employing different strategies to co-opt, hijack, or inhibit cellular processes. To restrict viral propagation and coordinate the immune response, different host cell receptors sense viral cues and trigger distinct antiviral signaling pathways driving the production of interferon (IFN), IFN-stimulated genes (ISG’s), and proinflammatory cytokines (Kreijtz et al., 2011; Chen et al., 2018). To usurp these antiviral pathways, viruses can exploit different characteristics of RNA molecules, including their chemical modifications. These RNA modifications, also known as the epitranscriptome, are catalyzed by different classes of RNA-modifying enzymes, occur in both host and viral RNAs, and have a direct impact on RNA maturation, stability, transport, and translation (Shelton et al., 2016; Boo and Kim, 2020; Xu L. et al., 2021; Boccaletto et al., 2022; Cui et al., 2022; Wilkinson et al., 2022; Zhang Y. et al., 2023). Notably, impairment of RNA modifications due to disruption of RNA modifiers has been shown to rewire the cellular epitranscriptome, and thus gene expression, being implicated in different pathologies (Huang et al., 2020). Although most studies are centred on ribosomal RNA (rRNA), transfer RNA (tRNA), and messenger RNA (mRNA) modifications, emerging evidence also emphasizes their relevance in other non-coding (nc) and long-nc (lnc) RNAs (Squires et al., 2012; Amort et al., 2013; Sun et al., 2021a; Ge et al., 2021). Several epitranscriptomic marks identified on viral RNAs facilitate viral propagation (Gokhale et al., 2016; Courtney et al., 2017; McIntyre et al., 2018; Ringeard et al., 2019; Tsai et al., 2020). On the other hand, reprogramming of host RNA modifications is correlated with coordination of antiviral responses (Rubio et al., 2018; Winkler et al., 2019; Lou et al., 2021). However, despite the growing understanding of the relevance of the RNA epitranscriptome during viral infections, and its potential as antiviral target, the field is still largely underestimated. In this review, we delve into how viral and host RNA epitranscriptomes impact infectious disease pathogenicity by shaping virus-host interactions.

## Unveiling the role of viral mRNA modifications during virus infection

To facilitate their replication and elude host immune surveillance, viruses evolved several tactics, including the modification of its own viral RNA molecules. The repertoire of epitranscriptomic modifications in viral mRNAs is expanding and, in the next sections, we discuss how viruses introduce epitranscriptomic marks into their own mRNA to potentiate their replication.

### 2′-O-methylation and 7-methyguanosine

mRNAs shield their 5′ end with an inverted N-7 methyl guanosine nucleoside (m^7^GpppN, cap1), along with an 2′-O-methyl group (cap2) within the first and second nucleosides downstream of the m^7^G cap (Pestova et al., 2001; Gebauer and Hentze, 2004; Chu et al., 2011; Varela et al., 2014; Hyde and Diamond, 2015; Devarkar et al., 2016). This capping event regulates several RNA functions, including metabolism, stability, and discrimination between self and foreign RNA (Hyde and Diamond, 2015; Ramanathan et al., 2016; Schlee and Hartmann, 2016; Decombe et al., 2023). Indeed, while cellular mRNAs with caps evade immune responses, pathogenic RNAs lacking caps are recognized by cellular sensors, triggering the activation of the IFN response (Züst et al., 2011; Mersinoglu et al., 2022).

Viruses evolved different strategies to incorporate a modified cap onto viral mRNAs (Hyde and Diamond, 2015; Sacco and Horner, 2021; Decombe et al., 2023). Influenza A virus (IAV), for instance, utilizes a cap-snatching mechanism to hijack m^7^G caps from host RNAs, allowing it to compete with host mRNAs for the translation machinery (Gu et al., 2015; De Vlugt et al., 2018). In contrast, certain viruses have evolved cap-independent modes of translation, utilizing internal ribosome entry sites (IRES) or other RNA structural elements that foster translation initiation (Hao et al., 2022). Several viruses encode specific methyltransferases that methylate the 2′-O-position of the ribose sugar of viral RNAs (Sorokin et al., 2021). Coronaviruses exploit their own NSP14 and NSP16 activities to introduce N-7 and 2′-O-methylation, respectively, into the viral mRNA (Chen et al., 2011; Chen et al., 2013), whereas dengue virus (DENV) and Ebola virus cap methylation relies on the activity of NS3, NS5, and L proteins, respectively (Daffis et al., 2010; Züst et al., 2011). Importantly, defects in the methyltransferase activity of these proteins is described to enhance the IFN response (Züst et al., 2011; Chang et al., 2016; Ringeard et al., 2019). The VP39 protein of vaccinia virus methylates viral 5′ caps to escape recognition by IFN-induced RNA binding protein 1 (IFIT1) (Daffis et al., 2010; Züst et al., 2011; Kumar et al., 2014; Menachery et al., 2014). Besides this, the human immunodeficiency virus 1 (HIV-1) was shown to hijack the host 2′O-MTase RNA 2′-O-methyltransferase 3 (FTSJ3) - Transactivation response RNA binding protein (TRBP) complex to modify its own RNA genome and escape recognition by the ISG 20-kDa (ISG20) protein (Ringeard et al., 2019; El Kazzi et al., 2023).

### N^4^-acetylcytidine

In humans, N^4^-acetylcytidine (ac^4^C) marks are found within tRNA, rRNA, and mRNA molecules (Thomas et al., 1978; Boccaletto et al., 2022), to enhance their stability and function in translation (Arango et al., 2018). ac^4^C is introduced onto RNA molecules under the actions of N-acetyltransferase 10 (NAT10), which requires the assistance of THUMP domain-containing 1 (THUMPD1) (Sharma et al., 2015; Broly et al., 2022) and box C/D snoRNA U13 (Sharma et al., 2015), to introduce the mark into tRNAs and rRNAs, respectively (Xie et al., 2023).

ac^4^C was identified in the genomes of Zika virus (ZIKV), DENV, hepatitis C virus (HCV), poliovirus (PV), HIV-1, enterovirus-71 (EV71) and IAV (McIntyre et al., 2018; Tsai et al., 2020; Furuse, 2021). HIV-1 and EV71, for instance, co-opt the host’s NAT10 to acetylate their transcripts at multiple sites to enhance viral gene expression (Tsai et al., 2020; Hao et al., 2022). The ac^4^C modification of EV71 allows the recruitment of poly (rC)-binding protein 2 (PCBP2) to the EV71 IRES, enhancing transcript stability and the interaction with RNA-dependent RNA polymerase (3D) (Hao et al., 2022).

### Pseudouridine

Pseudouridine (*Ψ*) is one of the most prevalent RNA modifications, and a substantial amount of this modification has been found in the genomes of several viruses, including ZIKV, DENV, HCV, PV, IAV and HIV-1 (Durbin et al., 2016; McIntyre et al., 2018; Furuse, 2021; Martinez Campos et al., 2021). Notably, *Ψ*-modified small RNAs derived from HCV were shown to bind to RIG-I with high affinity, but failed to trigger the canonical RIG-I conformational changes associated with its activation (Durbin et al., 2016). Furthermore, in a CRISPR screening seeking for host cell factors targeting HCV and DENV, several *Ψ* synthases were identified (Marceau et al., 2016).

Recent research has further revealed the involvement of *Ψ* in the regulation of alternative splicing (Karijolich et al., 2015). Notably, in betacoronaviruses such as SARS-CoV-2, *Ψ* was shown to significantly impact the splicing patterns of betacoronavirus-associated genes (Karlebach et al., 2022). Moreover, *Ψ* has also been showcased as an important element in Epstein-Barr virus (EBV) infections, with one of its non-coding RNAs, EBV-encoded RNA 2 (EBER2), being significantly marked by pseudouridylation (Henry et al., 2022). Disruption of pseudouridylation in EBER2 has been associated with decreased RNA levels and reduced efficiency in viral infection and the viral lifecycle (Henry et al., 2022).

### 5-methylcytidine

5-methycytidine (m^5^C) is found within multiple RNA classes and is catalyzed by enzymes belonging to the NOL1/NOP2/SUN domain (NSUN) family and DNA methyltransferase family protein (DNMT2) in eukaryotes (Bohnsack et al., 2019).

Accumulating evidence indicates that several viruses harbor m^5^C on their genomic RNA (Eckwahl et al., 2020; Cristinelli et al., 2022). For instance, m^5^C marks on murine leukemia virus (MLV) transcripts are recognized by the m^5^C reader ALYREF to promote their nuclear export (Eckwahl et al., 2020). The host’s RNA methyltransferase NSUN2 was shown to introduce m^5^C in HIV-1 genomic RNA to regulate viral gene expression. In accordance, depletion of NSUN2 reduced the abundance of m^5^C in HIV-1 transcripts and inhibited viral propagation by disturbing the splicing and translation of viral mRNAs (Courtney et al., 2019; Kong et al., 2020). Defects in NSUN2 activity also reduced the levels of m^5^C-enhancer RNA (eRNA), a transcriptional activator that, upon methylation, contributes to the metabolic reprogramming of HCV-infected cells (Shlomai et al., 2012).

Another RNA methyltransferase, DNMT2, has been shown to be translocated to stress granules to methylate HIV-1 RNAs (Dev et al., 2017) and to be involved in the fruit fly response to *Drosophila* C virus (DSV) (Durdevic et al., 2013). NSUN1 was also shown to restrict HIV-1 replication by inducing m^5^C methylation of TAR RNA (Kong et al., 2020).

### N^6^-methyladenosine

N^6^-methyladenosine (m^6^A) plays a role in regulating mRNA stability, splicing, and translation (Shi et al., 2022; Xue et al., 2022). This modification is catalyzed by a complex of proteins, including methyltransferase-like 3 (METTL3), METTL14, and RNA-binding motif protein 15 (RBM15), among others, while eraser enzymes fat mass and obesity-associated protein (FTO) and alkB homolog 5 (ALKBH5) revert m^6^A marks (Xue et al., 2022).

Several m^6^A marks have been identified in the genomes of several viruses, potentially serving as a shield to avoid recognition by the immune system and IFN production (Li et al., 2017; Ye et al., 2017; Hesser et al., 2018; Imam et al., 2018; Gokhale et al., 2020; Tsai et al., 2020; Lu et al., 2021; Zhuang et al., 2023). For instance, during vesicular stomatitis virus (VSV) infection, METTL3-mediated m^6^A modification reduced production of viral dsRNAs to elude RIG-I or MDA5 detection (Qiu et al., 2021). Similarly, rotavirus (RV) infection potentiates m^6^A modifications on mRNAs by downregulating the levels of the m^6^A eraser ALKBH5 (Wang et al., 2022). In HBV infection, the viral protein HBx interacted with the METTL3/14 complex to affect the m^6^A content of viral RNAs, with m^6^A modification of the HBV epsilon stem-loop of pgRNA preventing its ISG20-mediated degradation (Imam et al., 2018; Kim and Siddiqui, 2021). In this context, 3-deazaadenosine (DAA), an m^6^A inhibitor, or inactivation of METTL3, hindered IAV replication (Bader et al., 1978; Fischer et al., 1990; Fustin et al., 2013; Courtney et al., 2017).

The cellular m^6^A machinery can have diverse regulatory roles during viral infections. For instance, the m^6^A reader YTHDC1 was shown to bind to HIV-1 transcripts in a METTL3-dependent manner to ensure effective splicing and viral production (N’Da Konan et al., 2022). YTH N^6^-methyladenosine RNA binding protein 1 (YTHDF1) was also suggested to facilitate EBV viral RNA decapping and promote its RNA decay by recruiting RNA degradation complexes (Xia et al., 2021).

### A-to-I RNA editing

A-to-I RNA editing is a post-transcriptional modification involving the conversion of adenosine (A) to inosine (I) within RNA transcripts (Pfaller et al., 2021) by a family of enzymes known as ADARs (adenosine deaminases acting on RNA) encoded by three genes in mammals: ADAR1 (ADAR), ADAR2 (ADARB1), and ADAR3 (ADARB2) (Slotkin and Nishikura, 2013; Zhu et al., 2023). A-to-I RNA editing can be found in both viral and host RNAs, shaping diverse aspects of virus-host interaction. Regarding viral RNA, editing can lead to sequence variations that impact viral replication, translation, and immune evasion (Pfaller et al., 2021; Zhu et al., 2023). ADAR1 is believed to be co-opted by several viruses, including measles virus (Cattaneo et al., 1988), hepatitis D virus (HDV) (Poison et al., 1996), HIV (Doria et al., 2009), IAV (de Chassey et al., 2013; Furuse, 2021), DENV (de Chassey et al., 2013), ZIKV (Khrustalev et al., 2017), and SARS-CoV-2 (Di Giorgio et al., 2020), to edit their own viral RNA. For instance, through an ADAR-1 mediated RNA editing event, an UAG stop codon in HDV RNA is recoded to UIG. As the latter is read as UGG, this recoding phenomenon leads to a translation read-through, resulting in the production of a protein involved in virus replication. ADAR1 has been described to edit RNA from HIV (Phuphuakrat et al., 2008) and measles virus (Ward et al., 2011), promoting and restricting their replication, respectively. Additionally, ADAR1 was shown to enhance DENV propagation by facilitating the translation of its non-structural proteins (de Chassey et al., 2013). The roles of A-to-I editing during virus infection has been extensively reviewed by others (Samuel, 2011; Thompson et al., 2021; Vlachogiannis et al., 2021; Song et al., 2022; Zhu et al., 2023).

## The host mRNA epitranscriptome: a new player regulating antiviral responses?

**FIGURE 1:**
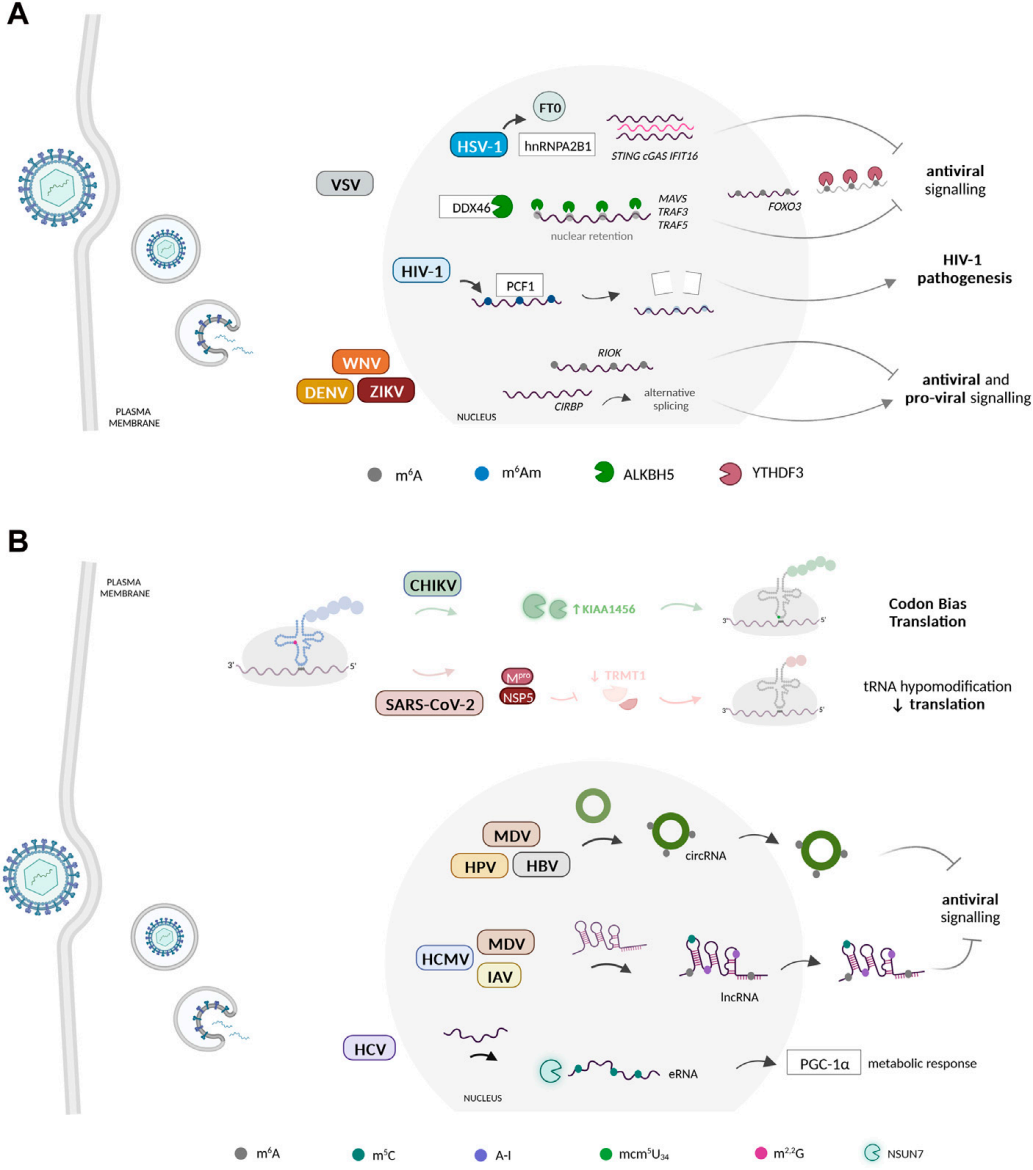
Dynamic changes in the host RNA epitranscriptomic landscape during virus infection. **(A)** Viruses reprogram host mRNA modification patterns to enhance their propagation. Several viruses have been shown to benefit from manipulating the m^6^A epitranscriptome of several host mRNAs. The host mRNA m^6^Am methylome was also recently proposed to play important regulatory roles during HIV-1 infection. These modifications are manipulated by viruses to regulate host gene expression during the infectious process. **(B)** Host non-coding RNAs undergo reprogramming at the epitranscriptome level during virus infection. Several viruses dynamically regulate the host ncRNA epitranscriptome to potentiate their replication, translation, and evasion from host immune surveillance. CHIKV induces a specific reprogramming of the host tRNA epitranscriptome via KIAA1456 and mcm^5^U_34_ to facilitate the decoding of its codon-biased transcripts. During SARS-CoV-2 infections, the levels of the tRNA-modifying enzyme TRMT1 and its modifications are significantly reduced. Beyond the tRNA, epitranscriptomic modification of other types of ncRNA molecules, including lncRNAs, circRNAs, and eRNAs, have been shown to play essential regulatory roles during virus infection, namely in the inhibition of the host antiviral response. Created with BioRender.com.

The host mRNA modification landscape undergoes substantial alterations during virus infection (Figure 1A). It is likely that viruses manipulate host mRNA modification patterns to rewire gene expression and hence modulate key biological processes undermining viral infections. For instance, it has been noted that m^6^A methylations are a known mark used by pattern recognition receptors (PRRs), to differentiate between host and foreign/pathogenic RNA. m^6^A-methylated RNA binds poorly to RIG-I, in contrast to RNA with Ψ which binds with high affinity, with both failing to initiate RIG-I downstream immune activation. In DNA viruses infections, disturbing hnRNPA2B1-FT0 interaction elevated the levels of cyclic GMP-AMP synthase (cGAS), stimulator of interferon genes (STING), and interferon gamma inducible protein 16 (IFI16) m^6^A-modified mRNA to bolster the antiviral response (Wang et al., 2019) (Figure 1A). Conversely, METTL3/14 depletion boosted the IFN-response (Rubio et al., 2018; Winkler et al., 2019; Li et al., 2021), whereas ALKBH5 was shown to demethylate the mRNAs of MAVS, TRAF3, and TRAF5 to limit IFN production (Zheng et al., 2017) (Figure 1A). The YTH N^6^-Methyladenosine RNA Binding Protein F2 (YTHDF2) reader enzyme binds to methylated RNA, reducing RIG-I binding, blocking RIG-I conformational changes and IFN transcription (Lou et al., 2021). However, the exact mechanism by which this suppression occurs remains mostly unknown (Durbin et al., 2016; Lou et al., 2021).

m^6^A also seems to play a role in regulating the translation of ISGs during antiviral response. Studies have shown that a subset of ISGs, including interferon induced transmembrane protein (IFITM1), appear to have their translation enhanced by m^6^A and its machinery, in addition to the fact that YTHDF1 also increases the expression of IFITM1 in an m^6^A-dependent manner (McFadden et al., 2021). Similarly, YTHDF3 hampered ISG expression by enhancing FOXO3 translation, an inhibitor of VSV replication (Zhang et al., 2019) (Figure 1A). Still within VSV infection, a TBK1-METTL3 axis enhanced IRF3 mRNA stability and translation through its m^6^A modification (Chen et al., 2022). The latter was also shown to regulate IRF7 and TLR9 mRNA stability to amplify the IFN-response to RV and EBV infection, respectively (Zheng et al., 2021; Wang et al., 2022). The m^6^A content of two specific host transcripts, RIOK3 and CIRBP, was reprogrammed in response to DENV, ZIKV, WNV and HCV. Functionally, m^6^A gain or loss in RIOK and CIRBP mRNAs facilitated their translation and splicing, respectively, to regulate virus infection (Gokhale et al., 2020) (Figure 1A). Moreover, in response to IFN stimulation, m^6^A and YTHDF1 were shown to increase ADAR1 levels (Terajima et al., 2021). Knockdown of YTHDF1 reduces the levels of IFN-induced A-to-I RNA editing, leading to the activation of dsRNA-sensing pathways and increasing the expression of various ISGs (Terajima et al., 2021).

Besides the immune response, m^6^A can regulate other biological processes during viral infections. For example, pseudorabies (PRV) exploits its US3 protein to reduce the m^6^A methylome as part of the PRV-induced metabolic dysfunction (Jansens et al., 2022; Yu et al., 2023). Reduced m^6^A on *a*-ketoglutarate dehydrogenase transcripts also hindered itaconate accumulation and contributed to mRNA decay during VSV infection (Liu et al., 2019). Furthermore, HBV infection altered the levels of m^6^A of PTEN transcripts and inhibited the IRF3 nuclear export (Kim and Siddiqui, 2021). The m^6^A methylation of the Kaposi’s sarcoma-associated herpesvirus (KSHV) ORF50 RNA enabled its binding to the m^6^A reader SND1 for ORF50 transcript stability (Baquero-Perez et al., 2019).

Apart from m^6^A, HIV-1 infection decreased the amount of N^6^,2′-O-dimethyladenosine (m^6^Am) modified host mRNAs by degrading the phosphorylated CTD Interacting Factor 1 (PCIF1), an inhibitor of HIV-1 transcription (Zhang et al., 2021) (Figure 1A). Additionally, in Newcastle disease virus (NDV) infections, expression of two accessory non-structural proteins, V and W, relies on RNA editing (Jadhav et al., 2020). In the case of ZIKV, host-induced RNA editing has a pro-viral effect (Zhou et al., 2019; Zhu et al., 2023). Notably, both isoforms of ADAR1 (p110 and p150) promote ZIKV replication by inhibiting the eukaryotic translation initiation factor 2 *a* (eIF2α) phosphorylation and IFN during immune responses (Zhou et al., 2019; Zhu et al., 2023).

## The non-coding RNA epitranscriptome landscape during viral infections

Viruses have been shown to manipulate and exploit several host ncRNAs to successfully propagate and evade the host immune response (Figure 1B). As previously mentioned, viruses manipulate host gene expression to maximize their replication and elude the antiviral response. Though most studies focus on mRNA, recent findings showcase that viruses also impact host ncRNAs, and even encode their own to perturb host antiviral responses. Next, we explore what is known regarding the importance of the ncRNA epitranscriptome in the context of viral infections.

### The role of the host cell tRNA epitranscriptome during viral infections

Viruses heavily rely on the host cell translation machinery, including the host tRNAs, to effectively translate their genomes (Pavon-Eternod et al., 2013; Stern-Ginossar et al., 2019; Nunes et al., 2020). Chemical modification of tRNAs, catalyzed by several tRNA-modifying enzymes, are essential for translation efficiency, namely when occurring within the tRNA anticodon loop region (Nedialkova and Leidel, 2015; Pereira et al., 2018; Tavares et al., 2021). Although the role of the tRNA epitranscriptome in viral infections remains largely unexplored, Chikungunya virus (CHIKV) was shown to induce changes in tRNA mcm^5^ wobble modification levels, through increased expression of the tRNA modifying enzyme KIAA1456, which facilitated the decoding of CHIKV-biased transcripts (Jungfleisch et al., 2022) (Figure 1B).

tRNA^Lys^UUU is used by HIV as a primer for reverse transcription. It has been found that both mcm^5^s^2^ wobble modification and ms^2^t^6^A modification at position 37 of tRNA^Lys^UUU favor its interaction with HIV’s nucleocapsid protein NCp7, which is essential for the primer recognition (Graham et al., 2011). These tRNA^Lys3^UUU modifications directly impact NCp7 binding and remodeling of the human anticodon stem and loop domain (hASL^Lys3^), as this protein exhibits a higher affinity for the modified hASL^Lys3^UUU compared to the unmodified human tRNA (Graham et al., 2011). This shows that modifications of htRNA^Lys3^UUU play a critical role in determining the recognition by NCp7 before the annealing of tRNA^Lys3^UUU to the viral genome as the primer for reverse transcription (Graham et al., 2011).

Reprogramming of tRNA modifications was also observed during Shewanella phage 1/4 infection of the marine bacterium *Shewanella glacialimarina* (Lampi et al., 2023)*.* Late-infection viral transcripts favoured GUA codons, which correlated with increased queuosine (Q) modification at the wobble position of the corresponding tRNA^Tyr^UAC. This suggests a potential correlation between phage codon bias and tRNA modification content (Lampi et al., 2023). On the other hand, TRMT1 and its modifications were reduced in response to SARS-CoV-2 infection, likely due to the activity of specific viral proteases (D’Oliviera et al., 2023; Zhang K. et al., 2023) (Figure 1B). Interestingly, a multiplex small RNA sequencing analysis of nasopharyngeal swabs showed significant variation of tRNA modification patterns among patients with distinct clinical manifestations of COVID-19 (Katanski et al., 2022).

Also, some tRNA modifications have been found in viral RNAs (McIntyre et al., 2018), raising the question of whether tRNA modifying enzymes also play a role in catalyzing viral RNA modifications that may be important for viral replication. Specifically, mcm^5^s^2^U modification was found in PV, whereas ncm^5^U was identified in RNAs of ZIKV, DENV, HCV and PV (McIntyre et al., 2018).

Though a direct link between viral infections and host antiviral responses is still missing, emerging data suggests that tRNA modifications may regulate the immune response. In fact, naïve T cells increase TRMT61A and TRMT6 levels to methylate specific tRNAs during transition to an active state (Liu et al., 2022). At the peak of proliferation, wybutosine and ms^2^t^6^a also decrease drastically to promote ribosomal frameshifting (Rak et al., 2021). As loss of wybutosine increases ribosomal frameshifting, this may explain the HIV-1 preference for proliferating T cells (Rak et al., 2021).

It is worth mentioning that tRNAs comprise an abundant source of tRNA-derived small RNAs (tsRNAs) that serve a variety of cellular regulatory functions (Oberbauer and Schaefer, 2018; Liu et al., 2021). Alterations in tRNA pools are often associated with tRNA-derived fragments (tRFs) generation in response to viral infections (Wang et al., 2013; Zhou et al., 2017; Choi et al., 2020). For instance, the tRNA demethylase ALKBH1 mediated the cleavage of tRNA-GluCTC to promote RSV replication (Choi et al., 2022). Then, it is plausible that the tRNA epitranscriptome influences tRF biogenesis during viral infections; host cells may counteract viruses by changing tRF patterns, or these are exploited by viruses to empower their own spread. Interestingly, tRFs also harbor chemical modifications, but in what extent these regulate tRF functions is unclear (Guzzi et al., 2018; Su et al., 2022).

### Beyond tRNAs: impact of epitranscriptomic marks on other ncRNA molecules

The influence of epitranscriptomic marks on various ncRNA molecules, besides tRNAs, is becoming a relevant focus of investigation. Studies on miRNAs, lncRNAs and circRNAs have shown that ncRNAs play important roles in the regulation of immune function and the occurrence and development of viral infections. For instance, loss of m^5^C in the EBV ncRNA 7SL has been shown to enhance its binding to RIG-I and, thus the IFN response (Zhang Y. et al., 2022). Furthermore, during EBV infection, m^5^C loss increased the levels of another EBV ncRNA, EBV-encoded RNA 1 (EBER1), likely indicating that this ncRNA is an Angiogenin target for m^5^C-dependent cleavage (Henry et al., 2020). On the other hand, introduction of Ψ into EBER2 enhanced its stability and promoted EBV lytic replication (Henry et al., 2022).

The function of miRNAs during virus infections has gathered significant attention (Girardi et al., 2018; Letafati et al., 2022; Ostrycharz and Hukowska-Szematowicz, 2022). While some viruses encode their own miRNAs, others can either suppress or hijack host miRNAs to disturb host immune-miRNA translation to potentiate propagation (Mishra et al., 2020). Conversely, hosts can also exploit their miRNAs to suppress viral replication. Despite the increasing evidence demonstrating the crosstalk between viruses and miRNAs upon infection, the relevance of the epitranscriptome for some of the observed miRNA alterations is still not known. However, different studies linked RNA modifications to miRNA biogenesis and degradation in cancer and plants, respectively (Yu and Chen, 2010; Shelton et al., 2016; Li et al., 2018; Han et al., 2021; Marceca et al., 2021; Mei et al., 2023). Although there is not enough evidence to back up this hypothesis, it is tempting to speculate that a crosstalk between the epitranscriptome and miRNAs may also occur during viral infections to, for instance, induce the degradation of antiviral miRNAs.

lncRNAs are generated by the host to counteract infection, but viruses themselves can also encode lncRNAs to counteract host activities, thereby regulating host immune responses, viral gene expression, and viral replication (Rossetto and Pari, 2012; Rossetto et al., 2013; Xu J. et al., 2021; Li et al., 2022). Some lncRNAs can act as molecular decoys, sequestering viral proteins or miRNAs, hence preventing their interaction with host factors pivotal for viral replication (Liu and Ding, 2017). Interestingly, the expression and tissue specificity of lncRNAs is regulated by their epitranscriptome (Jacob et al., 2017). Specific HCMV and MDV lncRNAs have been shown to harbor m^6^A marks to increase stability (Sun et al., 2021b; Lee et al., 2022). The amount of m^6^A-modified lncRNAs increases with MDV infection and is accompanied by increased expression of METTL14 and ALKBH5 (Sun et al., 2021b). Similarly, an hyperediting of the viral edited repeat-long (ERL) lncRNA occurred during MDV infection to downregulate IFN, particularly at the MDV lytic phase, and correlated with increased ADAR1 activity (Figueroa et al., 2016). Recently, m^5^C peaks were identified, following IAV infection, in several host lncRNAs, mostly associated with immune recognition and disease pathogenesis, possibly to regulate host responses to IAV by influencing the expression and stability of specific lncRNAs (Jiang et al., 2023) (Figure 1B).

Circular RNAs (circRNAs) have recently emerged as a class of ncRNAs involved in viral infection. Several viruses can alter the circRNA landscape of infected cells to modulate host gene expression (Xie et al., 2021). Notably, viruses have been shown to exploit the cellular machinery to generate viral circRNAs for their own profit, while hosts may also use these molecules to suppress viral replication (Zhang X. et al., 2022). The circRNAs epitranscriptome has also been linked to viral infections. For instance, the m^6^A methylome of specific circRNAs, allied to ErbB and insulin pathways, was altered by MDV infection and enabled immune surveillance escape (Sun et al., 2021b). The HBV HBx protein upregulates METTL3 to introduce m^6^A onto circ-ARL3 to assist its reverse splicing and biogenesis (Figure 1B). Ultimately, circ-ARL3 acts as a sponge for miR-1303, counteracting its inhibitory effect on specific oncogenes (Rao et al., 2021). Besides that, the oncogenic human papillomavirus (HPV) was shown to form circRNAs, including the m^6^A-modified E7 oncogene (circE7), which associated with polysomes and was translated into the E7 oncoprotein (Zhao et al., 2019).

Viruses can also affect the expression and function of other ncRNAs, such as small nucleolar RNAs (snoRNAs) (Stamm and Lodmell, 2019), piwi-interacting RNAs (piRNAs) (Wang et al., 2018; Joosten et al., 2021; Corsello et al., 2022; Wang X. et al., 2023) and eRNAs (Perez et al., 2012), to establish a favorable environment for viral propagation. Although not much is known regarding this altered expression and putative epitranscriptome reprograming, loss of m^5^C via NSUN7 depletion leads to hypomethylation of eRNAs, a transcriptional coactivator that interacts with proliferator-activated receptor-gamma co-activator 1 alpha (PGC-1α) to modulate cellular metabolic responses. As the metabolic response is reprogrammed during HCV infection, and accompanied by PGC-1α induction, NSUN7 may be involved in this process (Shlomai et al., 2012).

## Final remarks

The RNA epitranscriptome plays a critical role in shaping viral infections and host antiviral responses. It affects viral RNA stability, translation, and recognition by the host, hindering the host’s ability to detect viruses and mount a response (Gokhale et al., 2016; Courtney et al., 2017; Imam et al., 2018; Courtney et al., 2019; McFadden et al., 2021). Conversely, host RNA modification reprograming upon infection affects the expression of antiviral genes that suppress viral replication or facilitate viral gene expression (Rubio et al., 2018; Wang et al., 2019; Winkler et al., 2019; Wang H. et al., 2023).

A comprehensive understanding of the enzymes responsible for writing and erasing RNA modifications, as well as the reader proteins that interpret these modifications, presents exciting opportunities for developing more effective antiviral therapies. The development of small-molecule inhibitors, RNA-targeting therapies, and epitranscriptomic editing tools, holds promise in defeating viral resistance to antiviral therapies. While the field of RNA modification-targeted antiviral drugs is still in its early stages, several studies have shown promising results in potential targets. For example, in HSV-1 infection, inhibition of m^6^A by 3-DAA significantly decreased viral replication (Feng et al., 2022). Conversely, YTHDF1 recognition and destabilization of m^6^A-modified EBV transcripts, suppresses EBV infection, showing that this enzyme induces RNA deterioration (Xia et al., 2021). Nevertheless, several challenges lie ahead.

To grasp the crosstalk between viruses and the RNA epitranscriptome, a detailed mapping and characterization of RNA modifications during virus infection is required. Additionally, unraveling the crosstalk between different RNA modifications, their modifying enzymes, and their interplay with viruses will deepen our understanding of the complex dynamics at the virus-host cell interface. Further research efforts and technological advancements are crucial for fully harnessing the therapeutical potential of the RNA epitranscriptome.
